# Characterization of Damage Evolution on Hot Flat Rolled Mild Steel Sheets by Means of Micromagnetic Parameters and Fatigue Strength Determination

**DOI:** 10.3390/ma13112486

**Published:** 2020-05-29

**Authors:** Mirko Teschke, Julian Rozo Vasquez, Lukas Lücker, Frank Walther

**Affiliations:** Department of Materials Test Engineering (WPT), TU Dortmund University, Baroper Str. 303, D-44227 Dortmund, Germany; julian.rozo@tu-dortmund.de (J.R.V.); lukas.luecker@tu-dortmund.de (L.L.); frank.walther@tu-dortmund.de (F.W.)

**Keywords:** damage evolution and interaction, nondestructive characterization techniques, micromagnetic parameters, Barkhausen noise, incremental permeability, harmonics analysis, fatigue, electrical resistance, hot flat rolling, microalloyed mild steel, DP800

## Abstract

In continuous casting processes, inevitable voids (damage) are generated inside the material. The subsequent forming process of hot flat rolling offers the potential of healing these defects by closing the voids and bonding the internal surfaces. In this paper, different forming conditions from hot flat rolling process were characterized with micromagnetic measurement techniques and the influence of the damage evolution on the fatigue behavior was investigated. To characterize the reduction of voids through hot flat rolling processes, nondestructive testing techniques are required. Therefore, micromagnetic measurements such as Barkhausen noise, incremental permeability, and harmonic analysis were carried out, correlated with the number of voids, and compared with each other. The influence of damage evolution of different forming conditions on the fatigue behavior was characterized based on instrumented constant amplitude and multiple amplitude (load increase) tests. A significant increase in fatigue strength due to the hot flat rolling process, which leads to a reduction in the number of voids, was observed. In addition, the fracture surfaces of the specimens were analyzed in the scanning electron microscope.

## 1. Introduction

Nowadays, most of the steel used worldwide is produced via continuous casting and a subsequent rolling process. Even with the current technological progress, voids and shrinkage cavities inevitably occur in the material during continuous casting. The main causes of these are volume contraction and decreasing gas solubility during solidification [1]. Those can be considered as damage, which can be defined as the decrease in the load-bearing capacity due to the appearance and evolution of voids [2]. The forming process of hot flat rolling, i.e., high compressive stress at elevated temperatures, offers a potential of healing these defects by closing the voids and subsequently bonding the internal surfaces [3,4]. While healed voids have minor impact on fatigue behavior, closed voids could still leave a weak spot in the microstructure that may be prone to void nucleation during fatigue tests. These effects and their effectiveness depend on the various process parameters, especially the resulting load path [5]. 

During hot rolling, the load path is mainly influenced by the reduction in the pass [6]. Regarding [7], the pass reduction has a large impact on the stress triaxiality, which influences the void closure in the centerline of the slab. Small pass reductions cause more plastification on the surface of the slab, resulting in an increase in triaxiality in the center. On the other hand in Reference [6], the final thickness of the workpiece was identified as the predominating influencing factor on the void closure. With defined process parameters, not only the material geometry can be changed, but also the material properties and performance ability due to the dependency of the void closure.

Characterization of damage is a very complex task. In recent decades, the characterization of materials by means of nondestructive techniques has gained importance [8]. It is necessary to measure and control the material properties simultaneously to produce high-quality components. Metallographic examination with scanning electron microscopy (SEM), e.g., assessed by cross-sectional topography, is effective but time-consuming. Therefore, a reliable time-efficient and nondestructive test method is required to characterize the damage evolution and interaction during the manufacturing process.

Microstructural changes like dislocation density, phase content, and grain size, determine the mechanical material properties. Since the 1960s, these microstructural changes on steels are found to be correlated with parameters, like Barkhausen noise (BN) signal, coercivity, and upper harmonics, which are measured using micromagnetic analyses [9]. Some investigations show good correlations of the BN parameter with the residual stresses [10,11,12] and the changes of the hardness [13,14,15] in steel components. Microstructural changes like grain size [16,17], the evolution of crystallographic textures [18,19], and phase transformation [20,21] are correlated with the same parameter. Under cyclic loading of steel specimens, variations of the coercivity [4,22,23] and BN signals [24,25,26] have been characterized. Wear and forming-induced damage on steels are features that can be also investigated by micromagnetic testing [27,28,29,30]. Harmonic analysis has so far only used in [31] for damage characterization.

However, it is not only important to characterize the damage state but also to know the influence on the performance ability, especially on fatigue behavior and damage evolution. So far, only a few investigations have been made on the influence of forming-induced damage on the fatigue behavior [6,32]. Liebsch et al. investigated hot flat rolled steel and described an improvement of fatigue strength with a decreasing number of voids in fatigue tests in the low cycle fatigue (LCF) range and low test frequency. This effect was explained only in terms of damage, since interference of microstructural factors, such as grain size and hardness, could not be excluded [6].

This study focuses on the characterization of damage of microalloyed mild steel, before and after the hot flat rolling process. These analyses involve multiparameter tests, to extend the scope of the measurement techniques. This way, new possibilities for quality control are introduced, to produce property-optimized components based on a process–structure–property–damage understanding. Using micromagnetic test methods like Barkhausen noise, incremental permeability, and harmonic analysis, the damage state and evolution in the component will be characterized. The micromagnetic parameters are correlated with the number of voids, and the test methods are compared and evaluated concerning their suitability for damage characterization. Different damage levels set by varying process parameters on the fatigue behavior are evaluated in comparison to initial condition. Material response parameters, such as the deformation-induced strain and change in temperature or electrical resistance, are measured in situ during fatigue loading. This is intended to identify the characteristic parameters and their critical values that indicate failure at an early stage [33,34].

## 2. Materials

The material investigated in this study is a microalloyed structural steel with small additions of manganese, silicon, and chromium produced by continuous casting. In the initial condition (IC) the steel was not annealed after casting. However, the material has the potential to be transformed into DP800 in further process steps. The chemical composition is shown in Table 1.

In this study, four different forming states in addition to the IC were investigated. The specimens were produced at the Institute of Metal Forming (IBF) of the RWTH Aachen University. The initial workpieces with a dimension of 650 × 260 × 140 mm³ (length × width × height), were hot flat rolled on a universal rolling mill with a maximum rolling force of 4.0 MN and a maximum torque of 90 kNm. The material was hot flat rolled to two final thicknesses of t = 110 mm (forming condition 1, FC1) and t = 20 mm (forming condition 2, FC2). According to previous investigations, the pass reduction was identified as a critical parameter regarding void closure [7,35]. Therefore, two different process routes were chosen to reach the final thicknesses: one route with a pass reduction of 5 mm (Δh5) and the other one with a pass reduction of 15 mm (Δh15). The material is rolled to the final thicknesses in one heat with a starting temperature of 1200 °C.

**Figure 1:**
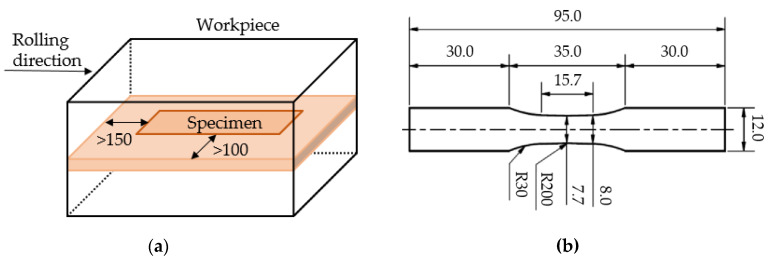
Specimen geometry for fatigue tests and micromagnetic investigations (in mm): (**a**) area for specimen extraction with respect to the rolling direction (RD); (**b**) geometry and dimensions adapted from [30].

Outside the center of the hot rolled workpieces, higher shear stresses are present in the material (compared to the center) [36]. This must be taken into account, as different strain states affect the void closure [37,38]. Therefore, all specimens were extracted from the middle section of the respective workpieces (IC, FC1, FC2) by wire-cut EDM, as shown in Figure 1a. The specimens were cut keeping clearances of 100 and 150 mm with respect to the edges on the rolling and transversal directions, respectively. This ensures largely homogenous conditions due to the plane strain compression conditions in the middle of the rolled part. The investigations of this paper, therefore, refer to the center of the hot rolled workpieces. The specimen geometry and dimensions, with a thickness of t = 2 mm are shown in Figure 1b. To test application-oriented surface conditions, the surface of the specimens tested in the fatigue tests was not further prepared. To reduce the influence of the surface roughness and residual stresses for micromagnetic testing, these specimens were finished by a grinding process with a grain size up to #4000 followed by two polishing processes for five minutes each with diamond suspensions with a grain size of 3 and 1 μm before testing and analyzing.

## 3. Methods

### 3.1. Micromagnetic Analysis

**Figure 2:**
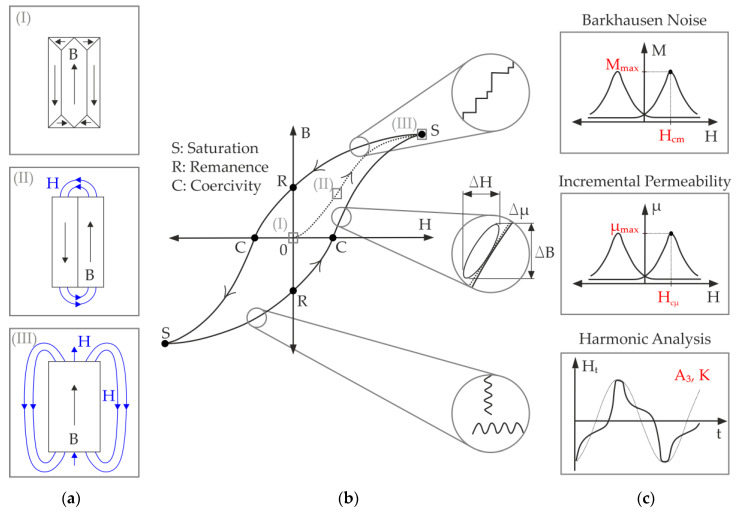
Magnetization of ferromagnetic materials: (**a**) evolution of magnetic domains; (**b**) hysteresis loop; (**c**) profiles of micromagnetic analysis techniques.

The continuous magnetization and demagnetization of ferromagnetic materials modify the magnetic domains, which are spontaneously magnetized regions [39]. An external magnetic field strength (H) causes a magnetic flux density (B) on the material and the movement of the domain walls, called Bloch walls, that divide the magnetic domains (Figure 2a). The cyclic process of magnetization is described through the hysteresis loop. Once the saturation (S) point is reached, the magnetization is no longer reversible. The coercivity (C) and remanence (R) are defined as the intercepts of the hysteresis with the horizontal and vertical axes, respectively (Figure 2b) [40,41]. From the hysteresis loop, different physical phenomena can be analyzed, which defines the three micromagnetic analysis techniques used in this investigation (Figure 2c).

#### 3.1.1. Barkhausen Noise (BN)

During the cyclic process of magnetization, the Bloch walls can collide with obstacles like lattice defects, voids, dislocations, and grain boundaries, which produce sudden jumps on the hysteresis curve, called Barkhausen noises. The amplification, filtration, and demodulation of the pulses allow the creation of an enveloping curve with respect to the magnetic field strength, to obtain the BN profile curve M(H), illustrated in Figure 2c. The highest density of these pulses is present on the coercivity point (C). This defines two important measuring parameters: the maximum of the M(H) curve, M_max_, and its corresponding magnetic field strength called the coercive magnetic field H_cm_ [9,42].

#### 3.1.2. Incremental Permeability (IP)

The IP is based on the eddy current measurement principle, which allows the characterization of conductivity and permeability of materials. A small high frequent field of the eddy current coil is superimposed by a low frequent magnetic field strength to create small reversible loops, which are overlapped on the hysteresis curve. The slopes of these mini loops are defined by Δµ = ΔB/ΔH and are dependent on the actual permeability on each hysteresis point. Hence, the impedance of the eddy current measurement coil changes along the hysteresis loop. The behavior of the IP Δµ in dependency of the low frequent exciting field defines the IP profile curve µ(H), as shown in Figure 2c. The magnetic field strength at which IP Δµ is maximal is the coercive magnetic field H_cµ_ [9,42].

#### 3.1.3. Harmonic Analysis (HA)

The HA is based on the analysis of the time-dependent signal of the tangential magnetic field in the specimen. The induced sinusoidal magnetic field is distorted by the different magnetic properties and features of the material. By Fourier analysis of the measured tangential magnetic field, a fundamental wave and the upper harmonics can be numerically obtained, which give information on the ferromagnetic properties at the surface of the material. Because of the symmetry of the hysteresis of magnetization, an odd number of harmonics is generated. In this investigation, the amplitudes *A*_3_, *A*_5_, *A*_7_ of the 3rd, 5th and 7th harmonics, respectively, are measured. From these, the harmonic distortion K is then determined according to Equation (1) [9,42,43].

(1) $$K\;[\%]=100\;\sqrt{\frac{{\left|A_{3}\right|}^{2}+{\left|A_{5}\right|}^{2}+{\left|A_{7}\right|}^{2}}{{\left|A_{1}\right|}^{2}}}\;$$

The micromagnetic measurements were carried out using the device 3MA II (micromagnetic multiparametric microstructure and stress analysis), manufactured by Fraunhofer IZFP (Saarbruecken, Germany), with the sensor SN18201. The magnetization frequency was set at 200 Hz and the magnetization amplitude at 80 A/cm. The magnetization phase offset was fixed at 70° and the sharpness parameter at a value of 15. The bandpass frequencies were filtered at 1 MHz for the lowpass, with a gain value of 5 dB. The measured parameters are summarized in Table 2.

**Figure 3:**
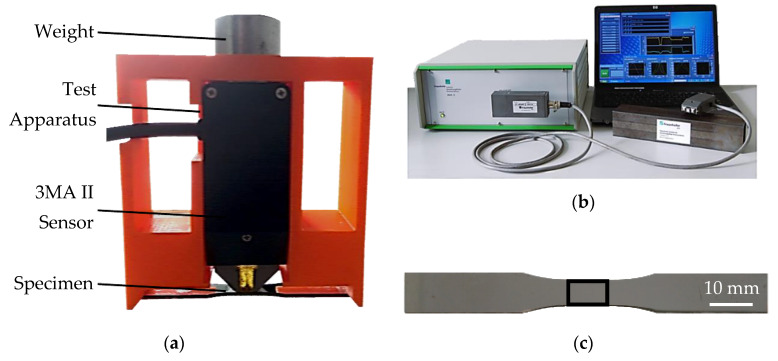
Micromagnetic analysis of hot flat rolled mild steel: (**a**) measurement setup; (**b**) 3MA II device by Fraunhofer IZFP; (**c**) specimen with measurement area.

For the micromagnetic measurements, a test apparatus using 3D printing (Figure 3a) was designed and applied successfully. This device ensures that the conditions and contact area between the sensor and specimen were always the same (Figure 3b). This way, the reproducibility and repeatability of the measurement is guaranteed.

### 3.2. Fatigue Investigations

**Figure 4:**
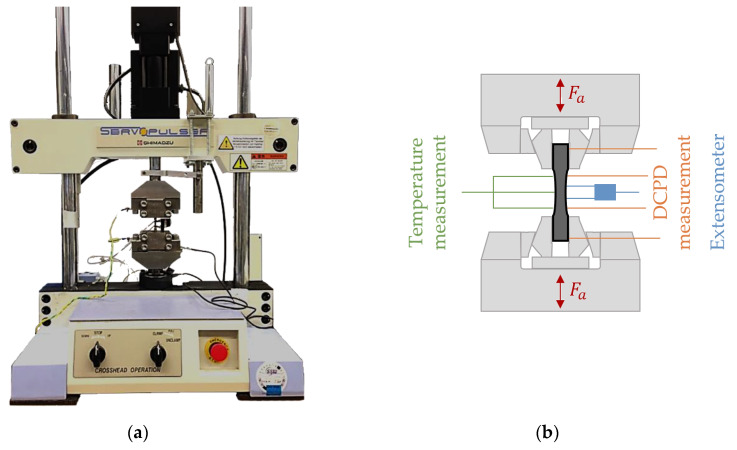
Test setup for fatigue tests: (**a**) servohydraulic testing system Shimadzu EHF-LV20; (**b**) schematic installation of the measuring technique.

For the characterization of the fatigue behavior, stress-controlled constant amplitude tests (CAT) and multiple amplitude (load increase) tests (LIT) with load-free intermediate steps were performed using the servohydraulic testing system Shimadzu EHF-LV20 (Shimadzu, Kyoto, Japan) which has a maximum load of F_max_ = ±20 kN (Figure 4a). The testing frequency of 10 Hz and the load ratio of R = 0.1 (tensile loading range) was used with a sinusoidal load-time function. All fatigue tests were performed at room temperature (RT) and a CAT run-out was defined reaching 2 × 10^6^ load cycles. 

In the LIT with load-free intermediate steps, the maximum stress is increased by Δσ_max_ = 20 MPa in each load step, starting from the starting stress of σ_max,start_ = 300 MPa. One step corresponds to 10^3^ cycles, which results in a time of 10^2^ s. After each load step, the specimen is unloaded (σ = 0 MPa) for the same time. This ensures that the deformation-induced heating of the specimen is decreased to zero to eliminate a temperature influence on the resistance measurements. 

The test setup is shown in Figure 4. During the fatigue tests, the strain was measured with the extensometer Sandner EXA-10-5 (l_0_ = 12.5 mm) manufactured by Sandner Messtechnik (Biebesheim, Germany). The plastic strain amplitude, as well as the total mean strain, are determined from the stress–hysteresis relationship recorded during the fatigue test with a self-developed LabView program. The specimen temperature was measured with thermocouples of the type K and the thermocouple input module NI 9210 (National Instruments, Austin, TX, USA). The electrical resistance of the specimen was determined in situ for each cycle at maximum stress (σ_max_). This was realized with the combination of a DC power source of the type Sorensen XTR6-110 (Ametek Programmable Power, San Diego, CA, USA) and an analog input module NI 9238 (National Instruments, Austin, TX, USA).

Fractographic images of the fracture surface were taken with the SEM Tescan Mira 3 XMU (Tescan, Brno, Czech Republic) with a working distance (WD) of 15 mm and an acceleration voltage of 15 kV.

## 4. Results and Discussion

### 4.1. Micromagnetic Analysis

As described before, ferromagnetic materials, like the investigated microalloyed mild steel, are composed of numerous magnetic domains divided by walls, called Bloch walls. The continuous magnetization and demagnetization process causes the motion of the Bloch walls [44]. Features like dislocations, voids, chemical inhomogeneities, second phase volumes, regions of inhomogeneous stress, and cracks are described as pinning sites. They have a defined critical value of the pinning force in the microstructure. Beyond this force, the Bloch walls break away from the pinning sites. The distribution and strength of the pinning sites hinder the free movement of the Bloch walls. Consequently, the measured micromagnetic signals are related to microstructural features [45].

**Figure 5:**
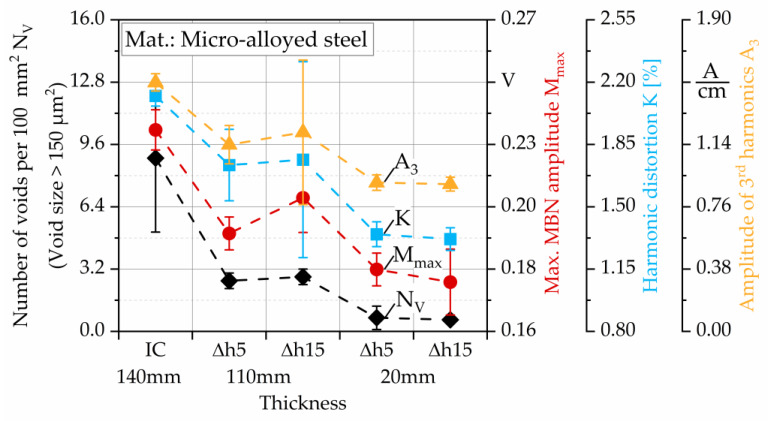
Maximum magnetic Barkhausen noise amplitude (M_max_), harmonic distortion (K) and amplitude of 3rd harmonics (A_3_), correlated with the number of voids >150 µm^2^ per 100 mm^2^ (N_V_) of hot flat rolled microalloyed mild steel. Number of voids from [6].

The process-related damage shown by the number of voids is correlated with the Barkhausen noise parameter M_max_, the upper harmonic amplitude A_3,_ and the harmonic distortion factor K. The number of voids with a size greater than 150 µm^2^, was determined by means of cross-sectional topography analyses in [6]. In previous investigations, it was found for steel DP800 that before heat treatment, the upper harmonic amplitude A_3_ is the most suitable parameter for damage characterization compared to higher harmonic amplitudes [30,31]. The evolution of the curves in Figure 5 is quasi-simultaneous, describing an appropriate correlation of the micromagnetic parameters and the damage evolution. The specimen thickness is t = 140 mm in the initial condition (IC), while in the hot flat rolling process the thicknesses are reduced to t = 110 mm (FC1) and t = 20 mm (FC2), resulting in the elimination of voids through mechanical closure according to [46]. The change in the microstructure is detected during the magnetization process by means of the mechanisms described before. The lower the number of voids (damage), the lower the distortions created by the motion of the Bloch walls, because less pinning sites are present in the microstructure. This leads to a decrease in M_max_ value. As the number of voids is reduced during hot flat rolling, the distortions of the induced tangential magnetic field decrease. This is reflected in lower amplitudes of the upper harmonics, especially A_3_ and the subsequent harmonic distortion K. Concurrently, A_3_ and K show a significant sensitivity to these microstructural changes, as also demonstrated in [31]. This way, harmonic analysis is also suitable to characterize damage.

**Figure 6:**
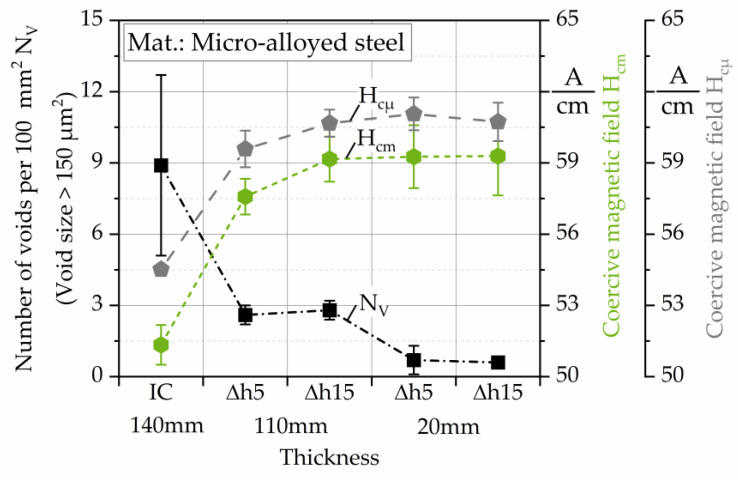
Coercive magnetic field by Barkhausen noise (H_cm_) and by incremental permeability (H_cµ_), correlated with the number of voids >150 µm^2^ per 100 mm^2^ (N_V_) of hot flat rolled microalloyed mild steel. Number of voids from [6].

The micromagnetic analysis of the coercive magnetic field shows similar results for the measurements performed by Barkhausen noise and by incremental permeability. The change of these parameters for the different rolling conditions exhibits an increasing trend with respect to the decrease in the number of voids (Figure 6). The opposite behavior of the coercive magnetic fields and M_max_ have been shown in previous works [14,47,48]. The minimum values of H_cm_ and H_cµ_ are present at the IC. After the hot flat rolling of the specimens, there is an increase in the values of these parameters.

On the measured micromagnetic parameters, damage is the dominant effect over residual stresses and dislocation density for two reasons: the void content in the IC and the surface preparation of the specimens. In the IC there are around 9 voids per 100 mm^2^ and they are reduced to 2.7 voids after the first hot flat rolling pass. This means a decrease of 70%, which makes the initial void content very large and the dominant effect of the BN measurements. Another fact is that the surfaces of the measured specimens were ground and polished. This makes negligible the effects of residual stresses, according to Reference [18]. The influence of hardness on the results can be also excluded. The hardness values measured in Reference [6] for the five states do not differ significantly as they are around 189 ± 6 HV10. The reduction in thickness produces changes in grain size. According to [4], the coercivity is linearly correlated with the grain size and the nonmetallic inclusions. However, the grain size measured in [6] does not correlate significantly with the micromagnetic parameters. The influence of the voids and the process-induced phase transformations could be reasons hiding this effect.

Thus, a nondestructive test method was validated in order to characterize deformation-induced damage evolution in the form of voids in a time-efficient way, to replace destructive testing with cross-sections. This nondestructive micromagnetic method is particularly interesting for application directly after the rolling process, in terms of property and quality control.

### 4.2. Fatigue Investigations

**Figure 7:**
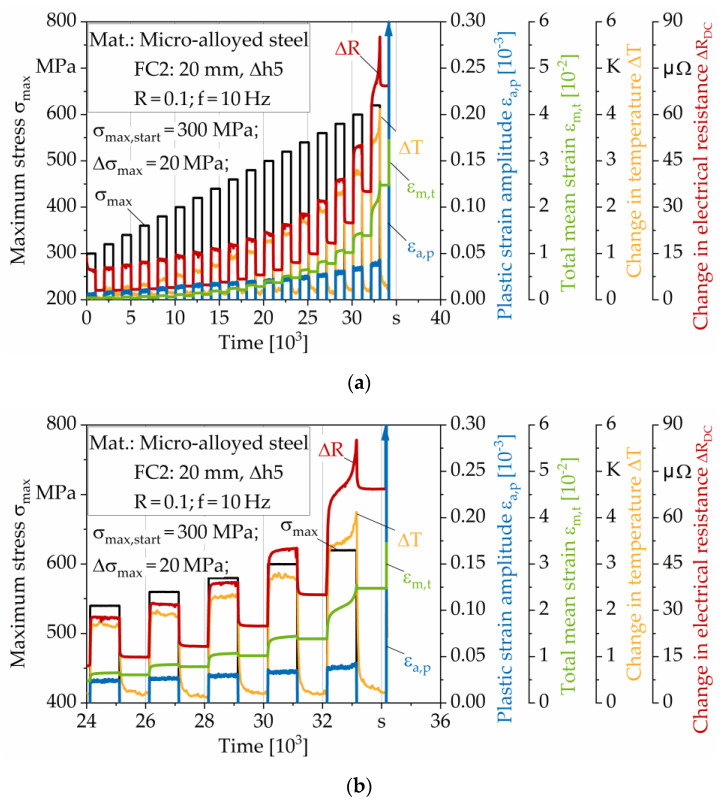
(**a**) Multiple amplitude (load increase) test (LIT) with load-free intermediate steps for hot flat rolled sheet material; thickness: 20 mm; pass reduction: Δ5 mm. (**b**) Scaled to last load steps.

Figure 7a shows an exemplary stepwise multiple amplitude (load increase) test with load-free intermediate steps on a specimen taken from a hot flat rolled sheet with a thickness of t = 20 mm and a Δh5 pass reduction. In addition to the stepwise increased maximum stress as the controlled variable, the total mean strain, plastic strain amplitude, change in temperature and change in electrical resistance are shown as the material response in the diagram. In Figure 7b, the last load and unload steps until failure are shown in detail. The specimen failed at the maximum stress of σ_max_ = 640 MPa. The courses described in the following are qualitatively transferable to the other forming conditions.

In both diagrams, it can be seen that the total mean strain ε_m,t_ increases with each load step. In the load-free steps (σ = 0 MPa) ε_m,t_ does not decrease to its value in the previous step. This phenomenon is known as cyclic creeping and “ratcheting”, respectively [49]. Since most loading energy dissipates into heat [50], the temperature increases with increasing stress. Within the load steps, the change in temperature ΔT increases progressively, while in the load-free steps, ΔT decreases regressively and approaches ΔT values nearby 0 K. The electrical resistance is influenced by geometry (length, cross-sectional area), defect density (dislocation structure/density, voids, etc.), and temperature [50,51,52]. Although the change in temperature decreases to ~ 0.1–0.2 K at the end of each load-free step, the change in electrical resistance ΔR_DC_ does not decrease to minimum values of ~mΩ. Therefore, the significant change in resistance during the LIT is not primarily influenced by the specimen heating, but by the geometrical specimen change, such as the reduction in the cross-sectional area and increasing length due to cyclic creeping, and the growth and unification of voids due to damage evolution. The progress of the change in resistance correlates with the total mean strain for loading and unloading steps. Consequently, electrical measurements have shown to be considerably applicable for this application of a hot flat rolled steel to characterize the influence of cyclic creeping and void evolution.

As a result of the stepwise increase in stress, the plastic strain amplitude and thus the cyclic strain hardening also increases with each step. Within the steps, the plastic strain amplitude does not increase significantly. As is typical for the stress ratio of R = 0.1 in tension loading range, the effect of cyclic creeping dominates in the form of total mean strain development. While the curves for ε_m,t_, ΔR_DC_, and ΔT show degressive progress within the steps until and including the stress step of σ_max_ = 600 MPa, there is an exponential increase in the three characteristic values in the stress step of σ_max_ = 620 MPa. In the first cycles of the stress step of 640 MPa, the specimen fails. Furthermore, the curve of ε_a,p_ shows an exponential increase within the stress step of σ_max_ = 620 MPa.

**Figure 8:**
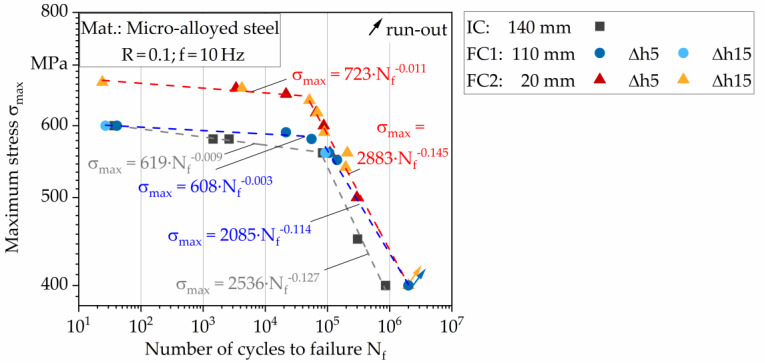
S-N curves for the initial condition and the different hot flat rolled conditions.

The data points from the CAT of the five different conditions were plotted in the form of an S-N diagram in double-logarithmic representation in Figure 8. Run-outs reached 2 × 10^6^ cycles. For t = 110 mm (FC1) and t = 20 mm (FC2) there is no significant difference between the pass reductions Δh5 and Δh15, as the data points are almost identical.

The S-N curves for FC1 and FC2 were therefore described using the same equation. The curves for the three different forming conditions (IC, FC1, FC2) can be each divided into two lines depending on the number of cycles to failure. Up to ~10^5^ cycles (low cycle fatigue, LCF), the line is very flat, but from ~10^5^ to 2 × 10^6^ cycles (high cycle fatigue, HCF) the line drops very steeply. For each line, the mathematical equation describing the course is given in Figure 8. The equation is composed according to Equation (2), in which the exponent n describes the slope of the straight line.

σ_max_ = C × (N_f_)^n^ (2)

The three straight lines run almost parallel in the LCF range, confirmed by a similar exponent. The IC and FC1 run on a similar level of maximum stress σ_max_. The reason for this could be that both conditions still have a large number of voids and a large void size (> 1000 µm) [6], which reduces the fatigue strength. In FC2, however, the number of voids and the void size (< 500 µm) is significantly reduced, consequently the S-N curve runs at a much higher level. Based on a number of cycles to failure of N_f_ = 10^4^ cycles, the fatigue strength of FC2 increases by 14% compared to IC, and the fatigue strength of FC1 only by 3%.

In the HCF range, the three states have a comparable slope, indicated by a similar exponent. The course of FC1 approaches that of FC2 for higher cycles. For the minimum stress of σ_min_ = 400 MPa, a run-out was measured for both FC. However, the IC fails already at 8·× 10^5^ cycles and thus only reaches about 40% of the maximum number of cycles. Based on the number of cycles to failure of N_f_ = 8 × 10^5^ cycles, the fatigue strength of FC1 and FC2 is 11% higher than that of the IC.

Since the hardness of the different conditions does not differ significantly with the thickness and the pass reduction, as discussed before [6], the difference in fatigue strength cannot be explained by production-related material hardening. Instead, a reduction in voids leads to a significant increase in fatigue strength. The results from the fatigue tests correspond to the fatigue tests of Liebsch et al. with load increase tests in the LCF range at a lower frequency of f = 1 Hz [6].

**Figure 9:**
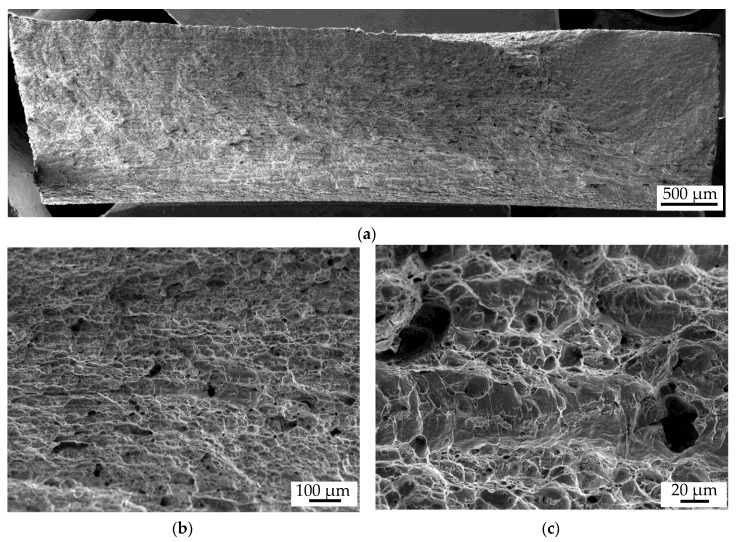
Fracture surface failed in constant amplitude test: σ_min_ = 500 MPa, t = 110 mm, Δh = 5 mm; (**a**) overview; (**b**,**c**) close-ups of voids.

Figure 9 shows fractographic SEM images of the fracture surface of a specimen tested at 500 MPa under constant amplitude loading with a thickness of t = 110 mm (FC1) and a pass reduction of Δh5. Figure 9a shows the whole fracture surface. The crack has been initiated at the specimen surface and a large sudden fracture can be observed, which can be deduced from the large area with a dimpled structure. The dimpled structure is even more visible in the close-up (Figure 9b). Additionally, voids, which were not completely closed and healed during the forming process, are visible on the fracture surface. The detailed image in Figure 9c shows that fatigue damage also initiates at voids. This can be detected by fatigue fracture surfaces between the voids. This reduces the cross-section and explains why specimens with lower number of voids have a higher fatigue strength. Although the voids do not lead to the initial cracking, they have a significant effect on fatigue strength. The observations of this exemplarily shown fracture surface are also transferable to the initial state as well as the other forming states.

In the VHCF range (N_f_ > 10^7^ cycles), the crack initiation in steels can occur at internal defects such as voids. Therefore, an even stronger influence of voids is expected in this lifetime range. Further investigations should be carried out in future to further determine the influence of damage evolution on the fatigue strength of semifinished parts in hot flat rolling, with the goal of understanding the process–structure–property–damage correlation in terms of separation and interaction of underlying damage mechanisms.

## 5. Conclusions and Outlook

In this paper, sheets of microalloyed mild steel from a hot flat rolling process were characterized by nondestructive micromagnetic testing methods of Barkhausen noises, incremental permeability, and harmonic analysis. All micromagnetic testing methods exhibited an excellent correlation with the number of voids and are therefore appropriate to characterize the damage reliably: the maximum amplitude of the magnetic Barkhausen noise profile (M_max_), the amplitude of the 3rd upper harmonics (A_3_), and the corresponding harmonic distortion factor (K). The coercive magnetic fields measured by Barkhausen noise (H_cm_) and incremental permeability (H_cµ_) manifest the opposite behavior, but with lower sensitivity to small changes. Thus, multiple nondestructive test methods were validated in order to characterize deformation-induced damage in the form of voids in a reliable and time-efficient method and to replace destructive testing with metallographic cross-sections. The evolution of damage has the dominant effect on the micromagnetic parameters because the number of voids in the IC is very large and the surfaces were prepared to make negligible the effect of roughness and residual stresses. In the future, sensor technology could be further developed for direct usage in the manufacturing process regarding quality and process control, based on an understanding of the process-structure-property-damage correlation in terms of separation and interaction of underlying damage mechanisms. To achieve this, the devices must be calibrated using destructive techniques like metallography, hardness measurements, and mechanical testing to perform quantitative analysis during production of components.

In constant and multiple amplitude (load increase) tests, the influence of damage on the fatigue behavior was investigated successfully. A significant difference between the fatigue behavior in the low cycle fatigue and high cycle fatigue ranges was found. The hot flat rolling process has significantly reduced the number of voids while increasing fatigue strength. The change in electrical resistance has proven to be a suitable parameter to describe the evolution of damage and cyclic creeping. This parameter could be used in the future as part of a condition monitoring system for components. While the cracks leading to failure are initiated at the specimen surface, fatigue cracks also occur at voids. This reduces the effective cross-section and supports failure. In the very high cycle fatigue range, crack initiation in steels can occur at internal defects, such as voids, therefore, an even stronger influence of voids is expected. Consequently, further investigations should be carried out in the future to understand the influence of process-induced damage on the fatigue strength of semifinished parts in hot flat rolling.

## Figures and Tables

**Table 1:** Chemical composition of the micro-alloyed mild steel, all data in wt% [6].

C	Si	Mn	Cu	Al	Mo	Ni	Cr	V	Nb	Ti	Co
0.1	0.4	1.8	0.05	0.03	0.02	0.05	0.2	<0.005	0.04	0.02	0.05

**Table 2:** Parameters measured in micromagnetic analysis.

Micromagnetic Analysis Technique	Parameter	Description	Unit
Harmonic analysis (HA)	A_3_	Amplitude of 3rd harmonics	A/cm
	K	Harmonic distortion	-
Barkhausen noise (BN)	M_max_	Maximum magnetic BN amplitude	V
	H_cm_	Coercive magnetic field by (BN)	A/cm
Incremental permeability (IP)	H_cµ_	Coercive magnetic field by (IP)	A/cm
